# Systematic Review and Future Direction of Neuro-Tourism Research

**DOI:** 10.3390/brainsci13040682

**Published:** 2023-04-19

**Authors:** Abeer Al-Nafjan, Mashael Aldayel, Amira Kharrat

**Affiliations:** 1 Computer Science Department, College of Computer and Information Sciences, Imam Muhammad Ibn Saud Islamic University, Riyadh 11432, Saudi Arabia; 2 Information Technology Department, College of Computer and Information Sciences, King Saud University, Riyadh 11543, Saudi Arabia

**Keywords:** neuroscience, neuromarketing, neuro-tourism, artificial intelligence, brain–computer interface, electroencephalography, eye-tracking, tourist emotion

## Abstract

Neuro-tourism is the application of neuroscience in tourism to improve marketing methods of the tourism industry by analyzing the brain activities of tourists. Neuro-tourism provides accurate real-time data on tourists’ conscious and unconscious emotions. Neuro-tourism uses the methods of neuromarketing such as brain–computer interface (BCI), eye-tracking, galvanic skin response, etc., to create tourism goods and services to improve tourist experience and satisfaction. Due to the novelty of neuro-tourism and the dearth of studies on this subject, this study offered a comprehensive analysis of the peer-reviewed journal publications in neuro-tourism research for the previous 12 years to detect trends in this field and provide insights for academics. We reviewed 52 articles indexed in the Web of Science (WoS) core collection database and examined them using our suggested classification schema. The results reveal a large growth in the number of published articles on neuro-tourism, demonstrating a rise in the relevance of this field. Additionally, the findings indicated a lack of integrating artificial intelligence techniques in neuro-tourism studies. We believe that the advancements in technology and research collaboration will facilitate exponential growth in this field.

## 1. Introduction

Tourism has attracted significant interest from academics since ancient times due to the great diversity of disciplines that went into its creation: geography, economics, psychology, marketing, history, culture, entertainment, and human social life. There is no denying the significance of tourism to the individual and economy. Through tourism, people gain new experiences, learn about other cultures, travel to new locations, engage in novel activities, restructure their everyday schedules, take in the beauty of nature or artificial aesthetics, attain self-relief, and improve their health state and quality of life [1].

Tourism has a significant impact on the economy by expanding employment prospects in transportation, travel agencies, restaurants, hotel administration, shops, and the export of domestic goods such as apparel, presents, souvenirs, and foods [2]. Additionally, developed nations have prioritized the tourism industry as a means of luring investors, raising income levels, generating employment possibilities, and promoting economic diversification.

Numerous research have demonstrated the influence of emotions (affective states) on tourists’ driving forces, actions, choices, and satisfaction in tourism and hospitality [3]. Measuring the feelings of tourists using subjective tools such as surveys and questionnaires is often insufficient. Tourism companies must look for clever ways to collect the objective measures of tourists as basic constructs to establish and expand their goods and services. Such objective measurements can be attained using neuro-tourism methods. Neuro-tourism is the application of neuromarketing in the tourism context to enhance tourist marketing strategies [4]. Neuromarketing recently appeared as “consumer neuroscience” [5], which integrates neuroimaging methods in the field of business to analyze customers cognitive, psychological, and emotional effects on marketing strategies.

Due to the novelty of neuro-tourism and the dearth of studies on this subject, this work intends to present a systematic assessment of peer-reviewed journal publications in neuro-tourism research for the past 12 years to identify trends in this field and provide insights for academics. It also seeks to provide an analytical framework (schema) for grouping and categorizing neuro-tourism papers for facilitating future studies on neuro-tourism.

This review helps to understand diverse applications, stimuli, measures, and associated ideas in the neuro-tourism field. As a result, this systematic review helps to serve as a reference tool for academics, researchers, and professionals who are interested in the tourism sector. The research questions that guided this work are:

1. Which psychological instruments, technological tools (measurements and sensors), and data analysis techniques are most commonly used to analyze tourist emotions and decision-making?
2. What are the limitations, challenges, and research lines that need to be addressed in developing and implementing technology platforms to investigate the behaviors of tourists in the neuro-tourism field?

This paper is organized as follows: Section 2 presents a background of neuromarketing; Section 3 describes the research methodology; Section 4 presents our suggested classification schema for neuro-tourism research; Section 5 illustrates the trends of the results derived from analyzing neuro-tourism articles based on the classification schema; and Section 6 discusses the results of the reviewed articles and the challenges and trends in neuro-tourism, as well as offers insights for future research; and, finally, Section 7 concludes the systematic review on neuro-tourism.

## 2. Background

**Figure 1:**
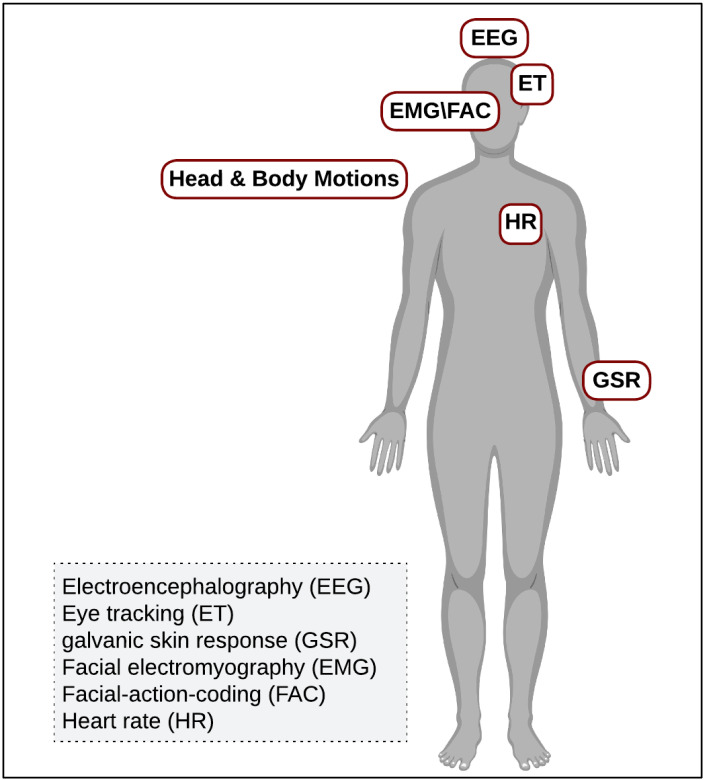
Most widely employed modalities in neuromarketing.

Neuromarketing makes use of many neuroscience methods to investigate consumers’ feelings and cognitive states. It uses two different modalities: (1) brain–computer interface (BCI) for either: (a) recording electrical brain activities such as EEG (electroencephalography), fNIRS (functional near-infrared spectroscopy), and MEG (magnetoencephalography) or (b) recording metabolic brain activities such as fMRI (functional magnetic resonance imaging) and PET (positron emission tomography); and (2) the recording of biological body activities such as galvanic skin response, facial-action-coding (FAC), eye-tracking (ET), and heart rate (HR) [6]. Figure 1 illustrates the common modalities in neuromarketing.

Each neuromarketing technique gathers different neural activity measurements. For example, fMRI assesses metabolic brain processes through variations in blood flow, while EEG records electrical changes in brain signals on the scalp [7]; ET measures eye attention toward a stimulus [8]; galvanic skin response gauges electrodermal activities through sweat production [9]; FAC measures feelings as expressions appear on the face; and HT counts the number of heartbeats in a time and can be used to determine stress [10,11].

Neuromarketing differs from traditional marketing techniques, such as audio advertisements, television advertising, surveys, and questionnaires, where the customer’s input is based on their thoughts and beliefs (subjective responses) [12]. This feedback does not provide company decision-makers with their customers’ real and unbiased opinions. However, incorporating neuroscience modalities into marketing plans will have an impact on gathering accurate emotional assessments for actual clients. This information will then be used to improve customer experience and enhance products and services based on their objective judgments [7].

**Figure 2:**
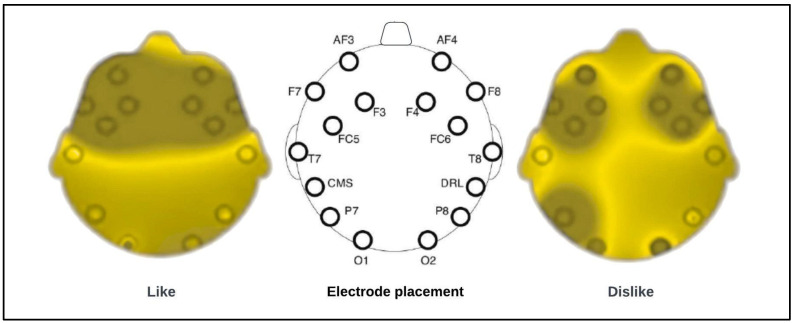
The correlation between EEG and decision-making of subjects.

For example, one of the most common neuroscience research methods that can be applied in a marketing context is EEG [7]. EEG brain signals would provide information regarding consumer preferences and behavior toward an advertisement where understanding the preferences of consumers will lead to higher returns and sales. EEG electrodes are placed on a participant’s head according to the international 10–20 system to measure the electrical activity in the cerebral cortex and to detect brainwaves. Then, the recorded brainwaves are translated using artificial intelligence and pattern recognition methods to recognize the participant preference, as shown in Figure 2.

Due to the enormous amount of data and ongoing increase in computing capacity, artificial intelligence (AI) and machine learning (ML) techniques have the potential to create business processes and improve their management in a variety of industry sectors, and the tourist industry is not an exception. The uncovering of the patterns of concealed relevant information from collected tourist data to improve tourism sector services and applications can be achieved with AI techniques [10]. Various modern AI technologies have been widely used in tourism and hospitality such as virtual reality, augmented reality, robotics, chatbots, and natural language processing [4]. However, research shows that there is also a need for the application of AI in neuro-tourism.

Neuro-tourism recently emerged as a branch of neuromarketing and in the use of neuroscience within the tourism industry [4]. Neuro-tourism can improve the research theories of tourism and hospitality. The same neuroscience techniques used in neuromarketing can be applied to neuro-tourism [10]. Additionally, high-dimensional data compiled from neuro-tourism investigations, including BCI recordings and psychobiological recordings, e.g., ET, can be fed to machines to build cutting-edge AI systems that anticipate traveler preferences or desires and thus generate individualized travel experiences [4].

## 3. Research Methodology

The research area of neuromarketing is composed of many scientific disciplines, such as computer science, psychology, social science, life science, and economics, and applied areas, such as tourism, management, finance, and engineering.

Although neuro-tourism research is a relevantly new and emerging topic, it has already attracted researchers from various disciplines and gained momentum as a field of academic research. Accordingly, a number of studies have been published in scientific journals and presented at conferences across major bibliometric databases such as Google Scholar, Scopus, and the Web of Science (WoS).

In our research, we opted to use the WoS Core Collection database as it contains peer-reviewed articles published in high-quality journals. We searched the WoS to conduct a thorough bibliographical search of the academic literature on neuro-tourism The scope of our study included articles related to neuroscience and the tourism industry. We utilized the basic search to locate subjects that fit our research parameter.

The inputs of the basic search were a combination of the keywords “tourism”, “neuroscience”, and “artificial intelligence”. We utilized substitute terms for the “tourism” keyword such as “tourist,” “travel,” “tour,” “destination”, and “hospitality”. We also substituted terms for the “neuroscience” keyword such as “neuromarketing”, “neuroscientific”, “BCI”, “eye tracking”, “skin conductance”, “EEG”, “electroencephalography”, “heart rate,” “fMRI,” and “emotion” or “affective computing.”

**Figure 3:**
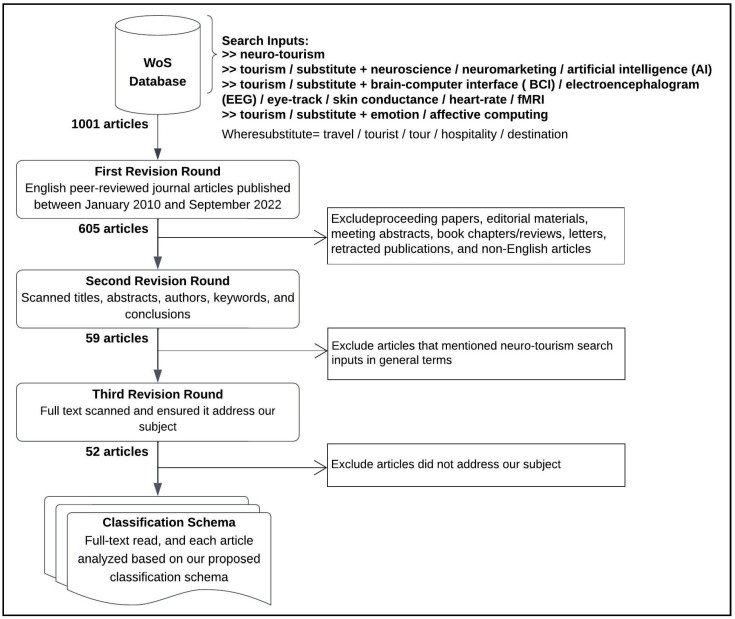
Research methodology.

Figure 3 shows our research methods, the review process, and filtering procedures. Initial search results returned 1001 items to which we applied our selection criteria for the first filtering process. For the selection criteria, we used only English peer-reviewed journal articles published between January 2010 and September 2022. We did not include proceeding papers, editorial materials, meeting abstracts, book chapters and reviews, letters, retracted publications, and non-English articles.

The first revision cycle ended with 605 search results. Then, we carried out a second screening procedure in which we manually reviewed each research paper to exclude non-neuro-tourism articles. In the second revision round, we scanned article titles, abstracts, authors, keywords, and conclusions and were left with 59 articles.

We added these articles to the online Zotero database to manage, evaluate, and review them. Finally, in the third revision cycle, we read the full text for the remaining 59 articles, and we eliminated 7 more articles because their topics were irrelevant to our theme. The remaining 52 articles were examined according to our classification schema.

## 4. Classification Schema

We created a classification schema for neuro-tourism research to systematically determine research insights in the field. Our classification design was based on gathering and examining the 52 research articles that came from the third revision round.

**Figure 4:**
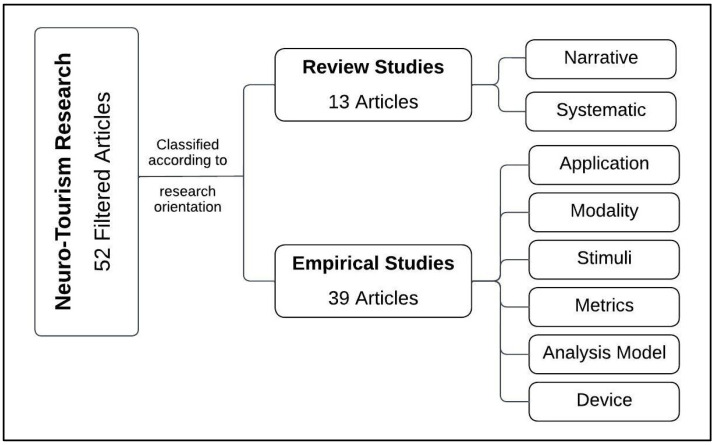
Classification Schema.

First, we divided them based on research orientation into review and empirical studies. Figure 4 displays our suggested categorization for neuro-tourism articles.

1. Review papers: Articles whose authors reviewed the literature of topics related to neuroscience in tourism and hospitality. These were further categorized into narrative and systematic reviews.

2. Empirical studies: Articles which covered tests conducted using at least one neuro-tourism technology such as ET, EEG, galvanic skin response, and others. We divided them further according to different subcategories: applications, modalities, stimuli, metrics, analysis models (methodology), and devices.

## 5. Results

**Figure 5:**
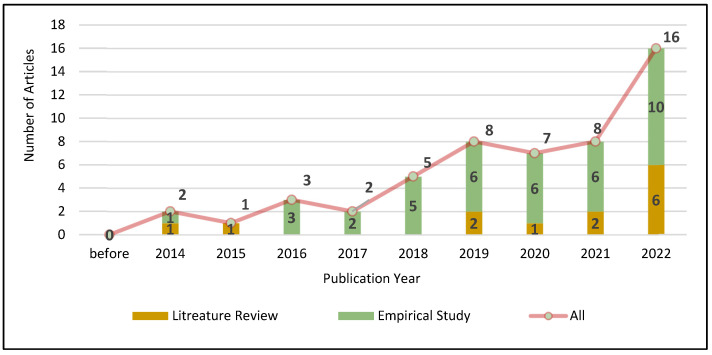
Trends in neuro-tourism research: number of articles by year of publication.

Figure 5 shows the number of articles published in the time frame between 2010 and 2022. The graph indicates that there were no articles in the field published prior to 2014. There were only two articles published in 2014: one review and one empirical research. This demonstrates the growth in neuro-tourism research which started from 2014 to 2022 for both literature reviews and empirical studies. We discovered that there were no empirical studies carried out during the years 2016, 2017, and 2018, and the concentration was on review research. Since 2018, the research has increased, reaching 16 articles by September 2022 (6 review research and 10 empirical studies). This demonstrates the growing interest in neuro-tourism and the value of research in the field.

We examined 52 neuro-tourism articles from 32 different peer-reviewed journals (See Appendix A for more information on the journals). We read, examined, and categorized each article according to our classification system. The ensuing subsections show the classification outcomes for both review articles and empirical research.

### 5.1. Review Papers

Our investigation found 13 review papers (25%) out of the 52 screened publications. Six articles were narrative reviews, and seven articles were systematic reviews. The following presents their research focus and direction.

For **systematic reviews**, Scott et al. [13], Atabay and Güzeller [14], and Savin et al. [8] assessed the application of ET in tourism research. Meanwhile, Li et al. [15] investigated the use of EEG in tourism and hospitality studies. Additionally, Doborjeh et al. [4] evaluated the relationship between AI methods and tourism and hospitality research. Moreover, De-Frutos-Arranz and López [7] looked at the application of neuroscience in tourism research. Finally, Lei et al. [10] covered the use of EEG, fMRI, and SC neurophysiological measures in hospitality and tourism research.

For **narrative reviews**, Koc and Boz [16] examined the psychological, neurological, biological, and chemical research findings in terms of their implications for tourism marketing. Li et al. [9] discussed the use of five measures (electro-dermal analysis, facial electromyography (EMG), HR, ET, and vascular measures) and their potential for the measurement of emotions for tourists. Skavronskaya et al. [17] investigated the association between novelty and memorable tourism experiences, while Tham et al. [18] examined the ethics in neuro-tourism research. Finally, Bastiaansen et al. [19] and Godovykh and Tasci [3] examined ideas in relation to emotions in tourism.

### 5.2. Empirical Studies

Out of the 52 filtered papers, we discovered 39 empirical studies (75%). We further divided them according to applications, modalities, stimuli, metrics, and analysis models. The ensuing sections show the outcomes.

#### 5.2.1. Applications

After we reviewed the empirical studies, we discovered that seven various applications have been applied in neuro-tourism: advertisement design, accommodation, tourist experience, restaurant experience, tourism risk, pricing, and destination. Table 1 lists these applications with the corresponding article counts and references. The following are details for these proposed applications based on our review.

##### Destination

Under the destination application, we included research on neuro-tourism tests that looked into the preferences for travel destinations and what affects tourists’ visiting intentions. In our review, we found 14 articles that investigated the formation of travel destination preferences. Bastiaansen et al. [21] undertook a neuromarketing experiment to determine whether a positive emotion toward a destination could be developed after inducing tourists to watch destination marketing videos. They measured the brain event-related potentials (ERPs) of 30 participants using EEG. They discovered that emotional responses toward a destination are greater after watching related destination videos.

To distinguish between different destination choices, Zoëga Ramsøy et al. [23] explored whether the direct emotional responses of visitors and the cognitive load toward various places may predict their stated preferences after monitoring them watching images and videos related to destinations using ET and EEG. They enlisted 32 individuals and discovered that combining subconscious measurements and conscious scores would improve forecasts of their actual decisions. Deng et al. [28] examined the impact of aesthetic perception influences on tourists’ intentions for destination choices. They evaluated visual aesthetics perceived by 64 individuals on destination images through the participants’ first impressions, visual appeals, and aesthetic emotions using ET. They concluded that there was a strong correlation between a destination’s aesthetics and the visiting intentions of tourists.

##### Advertisement Design

As tourism advertisements play an essential role in guiding customers, some of these advertisements failed and had little effect because of their poor design. Under the advertisement design application, we added neuro-tourism articles that explored the concept of improving the design of any kind of tourism advertisement, such as travel website design, A4 magazine design, multipage visitor guide design, or any design that focuses on tourism stimuli. In our review, we found seven papers discussing the type of advertisement design used for tourism commercials. The effectiveness of tourism advertisements on the emotions of tourists varies depending on the advertisement type. Kim et al. [34] compared watching video advertisements (multi-motion images with text) to hearing audio advertisements on the behavior of 42 participants. They gauged their attention through HR and arousal through ET. Findings show that attention in both advertisement types was the same, but participants’ arousals were higher in the case of video commercials. Participants also preferred video advertisements and demonstrated higher responses than audio advertisements.

Li et al. [35] investigated perceived advertising effectiveness in terms of visual attention toward tourism advertisements with text incorporated into landscape images. The visual attention of 37 participants was measured using ET, and information on perceived advertising efficacy was gathered using questionnaires. The effectiveness of text was investigated using understood versus non-understood language and single text messages versus multiple text messages. Higher attention was reported for images with text than without text, regardless of whether participants could understand the language. Participants viewed visuals with understood language for a longer time than with unidentified language. Additionally, images with single text were viewed for a longer time than images with several texts.

The effectiveness of travel agencies’ websites can also be evaluated to attract more tourists online. Muñoz-Leiva et al. [38] employed ET on sixty participants to test the effectiveness of the banner advertisement of three travel websites in terms of visual attention. The authors also evaluated participants’ memory recall using self-reports. They used the same banner advertisement with a fixed position on all three websites: a hotel blog, a Facebook profile, and a TripAdvisor profile. More effectiveness was found in Facebook profiles than in hotel blogs and the least in TripAdvisor profiles. The authors said that this is due to the simplicity of the Facebook editorial content available for companies and its well-organized elements.

##### Accommodation

Under the accommodation application, we incorporated neuro-tourism articles that discussed advertising tourist accommodations such as hotels or accommodations such as yoga and spa. We included articles that identified different effective elements contributing to the final consumer decision in choosing a place for lodging. In our review, we found seven papers that encouraged innovations in tourist accommodation.

With the help of brain activity and the monitoring (brain wave frequency measurements) of 16 participants using EEG, Hsu and Chen [40] investigated how hotel video advertisements, including those with happy face emojis, affect consumers’ choice of hotel. Eight videos selected from YouTube for prominent five-star hotels, including Marriott International and Sheraton, displayed their rooms presented with a smiling face emoji. Results showed an increase in beta and theta frequency bands, which indicates a strong beneficial influence on consumers’ hotel choices after watching hotel videos with a smiling face.

In a novel study, Fronda et al. [42] assessed how sustainability factors implemented by hotels improve consumers’ selection using three multi-modalities: galvanic skin response (GSR), HR, and EEG. In their experiment, nineteen participants explored four different areas of a green hotel which adopted a sustainability approach: halls, restaurants, bars, standard double rooms, and bedroom suites. Sustainability here means using eco-friendly items that do not have negative impacts on the environment. EEG results showed an increase in beta and theta frequency band activities, which supports the presence of cognitive and emotional responses. During the investigation of the hotel surroundings, the pulse volume amplitude and HR responses increased, showing emotional involvement and positive emotional reactions. As a result, applying a sustainability concept in accommodation services improves the satisfaction and personal experiences of tourists.

Recent research was conducted to promote hotels’ wellness offerings such as yoga and spa. For instance, Wang et al. [46] gathered 85 older consumers to evaluate the performance of eye movements while watching images of people practicing yoga in natural settings as well as those of man-made environments such as gyms. The images of wellness services with natural settings attracted more visual attention among older consumers and evoked a willingness to spend more time and money than the pictures with built-in environments.

##### Tourist Experience

In the application of tourist experience, we incorporated neuro-tourism articles that looked at the emotions of tourists when they were engaging in various tourism activities, including visiting gardens or heritage sites or riding roller coasters. Our research found six publications that examined feelings related to the tourist experience while engaging in various tourism activities.

Several tourists enjoyed trying adventure sports with benefits for the physical body, mental health, and better conduct. These adventurous pursuits can be demanding and stressful, such as rappelling (rock-climbing). Bailey et al. [47] investigated objective cognitive responses (anxiety, attention, and approach motivation) from ten participants while practicing in rappelling using a fitted EEG headset, and then compared it with their self-reported valence and arousal. Results showed significant increases in anxiety and approach motivation by EEG readings as well as strong relationships between self-reported valence and approach motivation as evaluated by EEG.

Many tourists are attracted to visiting historical landscapes. Li et al. [48] used EEG to study the attention perceived by participants toward four ancient Chinese gardens. Participants were divided into two groups. The authors acquired EEG readings for the first group with pictures of the gardens. The second group went to the gardens outdoors while the authors studied their EEG data. Findings showed a positive correlation between visitors’ attention and novelty, scene abundance, color sensitivity, and overall landscape harmonization in both groups. Results from outdoor visits revealed decreases in landscape attractiveness when the number of visitors rose.

##### Tourism Risk

Under this application category, we included articles that studied any type of tourism risk. Different types of tourism risks can happen in real life, including inclement weather, heavy traffic, low security, unhealthy food, inadequate medical care, and dirty lodging. In our review, we uncovered three publications that examined the connection between various tourism risks and the emotional reactions of tourists. These studies are crucial for travel businesses to offer safer services.

Wen [53] investigated the perception of tourism risk in an EEG experiment. He evaluated the brain ERPs of 17 participants to investigate various aspects of travel planning. He found a strong correlation between those who planned to travel and their choices due to individual preferences whether the time was suitable or not. Further, he discovered that people who frequently travel have a lesser perception of tourism dangers.

To examine the effect of insurance on tourists’ attention to risk and landscape, Wu [54] used an ET experiment on 105 participants carrying various travel insurance plans. He discovered that participants without insurance paid more visual attention to risk information than those with insurance. Participants with insurance also scored higher on a visual quality test than those without insurance.

##### Restaurant Experience

Food tourism inspires many travelers and contributes to their culinary and travel experiences. We discovered one article that looked into this phenomenon. Savelli et al. [56] looked at the ways to communicate typical–local cuisines to increase the appeal of culinary tourism. They assessed the visual attention and cognitive involvement of 40 participants using ET and EEG signals. They looked at three crucial dietary characteristics to determine which contributes to a higher attractiveness of typical–local foods: sustainability, geographical indications, or healthiness. The most alluring quality for visitors was the healthiness of regional cuisines, followed by geographical cues and sustainability.

##### Pricing

One of the key factors influencing tourist decisions is the price of tourism goods or services. We found one article that looked at the psychological effects of pricing on tourists. Boz et al. [57] conducted an experiment using four different neuromarketing modalities—GSR, ET, HR, and EEG—to shed light on how tourists perceive prices in holiday advertisements and pricing-related concerns in terms of design features, location, and content. This is advantageous for tourism and hospitality agencies when they need to include prices in advertisements.

The [57] study has various conclusions, some of which we discuss. They concluded that women paid more attention to discounted prices than men did. However, men’s attention to the discount rates was faster, and had more impulsive motivations. The authors found that consumers rarely read complete paragraphs describing the promotion of a product; instead, they choose to read short statements. Participants focused on the images with human faces and demonstrated greater attention to prices close to these faces. Additionally, female participants adopted unfavorable opinions regarding accommodation advertisements featuring female bodies. Last but not least, the study suggested focusing the visual promotion labels for installment plans, discount rates, and reduced prices.

#### 5.2.2. Modalities

We discovered numerous distinct methods for capturing human cognitive neural characteristics in neuro-tourism research: recording electrical brain activities such as EEG, recording physiological body activities such as ET, GSR, and facial measurements: either EMG or FAC, HR, and recording moving head and body activities. Table 2 provides the different modalities employed in neuro-tourism research and their sample size information.

ET was used most often; researchers used it alone in 21 experiments. For instance, using ET on 48 participants, Espigares-Jurado et al. [41] investigated how the primary images on hotel websites, typically found in the header, affect user visual attention patterns and cognitive processing. Additionally, Lever et al. [24] used ET with 22 participants to identify notable variations among readers of travel guidebooks to understand their reading behavior and provide destination marketers greater knowledge concerning the layout of their marketing materials.

EEG ranks the second most frequent modality used, employed alone in six trials. For example, Wen [53] used EEG to measure the ERPs of 17 participants to explore the various aspects influencing how visitors create travel plans. Bastiaansen et al. [51] also used EEG to examine the emotional reactions of 23 participants to images of places before and after watching travel commercials on television. Our research uncovered three studies that coupled ET with EEG.

As an illustration, Michael et al. [22] and Zoëga Ramsøy et al. [23] applied both modalities to 30 and 32 participants, respectively. Both research groups sought to comprehend the immediate, underlying emotional and cognitive reactions that are involved in the preferences for vacation destinations.

GSR is the third most common, used alone in two studies and five times in combination with other modalities such as EMG, FAC, EEG, ET, and HR. For instance, Di-Clemente et al. [33] used GSR on 38 participants to assess their immediate emotional reactions to images advertising travel locations. Boz et al. [57] also used GSR together with EEG, ET, and HR to examine how travelers view pricing in holiday advertisements. They used these integrated modalities to enhance the accuracy and dependability of data regarding how consumers see prices and pricing issues. FAC was used exclusively in one trial.

González-Rodríguez et al. [50] aimed to assess customer satisfaction with the level of service provided by the tourism industry. They used FAC to examine the instantaneous emotional responses of 230 people in a guided tour of a historical place. Lastly, head and body motions were used in one study along with ET and audio–visual data.

Matsuda et al. [49] conducted an experiment on 22 tourists walking on a path through different sightseeing places. The authors sought to create a model to assess the emotional well-being and degree of satisfaction of visitors. They used a SenStick device placed on the ear, which collects data on head and body movements. They also requested tourists to take selfie videos using their smartphones during the tour to gather information on their faces and voices. All four types of data were fed into a model to predict the tourists’ feelings while sightseeing.

#### 5.2.3. Stimuli

As senses play a critical role in how people interact with various buying and consuming activities, designing memorable experiences that captivate visitors on a deep level necessitates engaging their senses [59]. Therefore, creating the appropriate stimuli to engage all tourist senses positively is strongly advised. In our analysis, we found that studies have employed many stimuli to awaken tourists’ senses and gauge their emotional responses (see Table 3).

All neuro-tourism research that used various modalities used visual-based elicitation. For instance, images from the cities of Bruges and Kyoto were employed in the EEG study for the identification of travel preferences [21]. Additionally, in the GSR experiment, images of Egypt and the Caribbean were utilized with various typologies for the same goal—to identify preferences for locations [33].

Additionally, for restaurants, graphics with three different meanings (sustainability, geography, and healthiness) were generated to analyze the most appealing typical–local dishes presented by tourists [56]. In an experiment using ET, Wu [54] used images displaying risk information from Chinese online travel agencies to examine the impact of travel insurance on risk perception and vacation experience for visitors.

In several trials, images were also used with written text. For instance, Lin et al. [55] looked at the decisions of tourists in choosing adventure tourism items for themselves or as recommendations to others. They employed images with text indicating various risk levels from well-known Chinese travel websites for an ET experiment. In addition to using images with text, some experiments merged graphics with videos [22,23,31].

Some researchers discovered that evoking emotions using brief video snippets was effective. For instance, in an EEG trial, the use of hotel films with smiling face emojis had a favorable impact on the decisions of visitors [40]. In tourism advertisements, participants in [34] scored higher arousal when watching video commercials rather than hearing audio commercials. In addition to images and videos, visually based stimuli in neuro-tourism experiments included the use of text-only content [30,53], guidebooks [24,39], and websites [38,41,43,57].

The elicitation of emotions through planned tasks was mostly applied to the tourist experience. For instance, in the study by [42], participants were told to stroll through four distinct areas of a green hotel to gauge their level of satisfaction. Bastiaansen et al. [51] also used GSR to measure the emotional reactions of participants while experiencing a roller-coaster ride with or without virtual reality headsets.

Finally, according to Skavronskaya et al. [17], the more novel the stimulus, the more it influences memory. When several stimuli are shown, the one that differs from others is more likely to be recalled. Therefore, choosing the most effective stimuli in neuro-tourism investigations is critical.

#### 5.2.4. Metrics

In our review, we assessed several metrics using various modalities evoked by various stimuli. The choice of targeted emotion measurement depends on the case study. In general, our research found various emotional states mentioned in the literature: (i) affect, (ii) attention, and (iii) cognitive load as indicated in Table 4.

The concept of **affect** is related to emotion since affect is the outward manifestation of emotion. It consists of two dimensions: valence and arousal (dimensional model). Valence represents the positive, negative, or neutral state of emotion, while arousal denotes the intensity of the felt emotion [15]. Researchers evaluated affect using ET, GSR, EMG, and EEG. For instance, Bastiaansen et al. [31] used EEG to measure the valence and arousal of the participants to understand their emotional responses to destination images before and after watching a TV destination advertisement.

We discovered some research that examined certain emotions, including satisfaction [49,50], trustworthiness [45], anxiety [47], and aesthetic perception [26,28,48]. Wen [53] also determined preferences, which is the choice behavior using ERP in EEG tests. Preferences (choices) can be quantified based on the frontal asymmetry theory of brain activity [15].

**Attention** is also an emotional state representing the amount of focus on an object [7]. ET was the primary method for gauging consumer attention and the tool most used in advertisement designs. Several studies also used attention to identify other emotions. For instance, [26] measured the aesthetic perception of participants using visual attention (fixation count, fixation duration, and total time of visit).

**Cognitive load** is the amount of mental operations in the working memory [15]. It is used to test the amount of information proceeding or recalled as in the case of tourism advertisements. For instance, Espigares-Jurado et al. [41] employed ET to evaluate the effectiveness of the primary image within a hotel website. They measured both the attention and the working memory of the participants while browsing a hotel website. The cognitive effort was measured by fixation time, fixation count, and duration following task assignment.

#### 5.2.5. Analysis Model

In our analysis, we discovered many calculation techniques utilized to infer consumers’ emotional state from collected data. We separated these techniques into statistical and ML models. Table 5 provides a summary of these models and lists all preprocessing and feature extraction techniques found in the literature.

It is significant to note that the majority of the studies (69.23%) employed statistical analyses such as ANOVA, T-test, mean, *p*-value, and others to compute statistical features such as mean and standard deviation, whereas ML models were applied in 30.77% of the studies.

For classification analysis, we discovered one paper that used ML. Matsuda et al. [49] used RNN-LSTM to categorize three kinds of emotions (positive, negative, and neutral) to estimate visitor satisfaction. They achieved 0.48 of an unweighted average recall score in the classification of the three classes with a mean absolute error of 1.11 of a seven-level satisfaction estimation.

For regression analysis, we discovered 11 studies that used ML models. For instance, Zhao et al. [32] used multiple linear regression analyses with ANOVA to predict destination travel intention. They employed two features—fixation duration and fixation count—measured by ET for 120 individuals. They sought to determine changes in visit intention when changing either stimuli content (images of people vs. images of scenery vs. images of people and scenery) or stimuli type (scenery-only and text-only vs. text and scenery) with different positions (upper right vs. lower right vs. upper left vs. lower left). They found that tourists spent more time seeing stimuli that integrated scenery with text.

#### 5.2.6. Devices

In neuro-tourism research, as various modalities are used for recording human cognitive neural characteristics, empirical studies have employed different hardware devices for recording neuro-tourism data. Table 6 lists these devices with associated article counts and references. It is notable that the number of electrodes differs for EEG investigations. For instance, Bastiaansen et al. [21] used an elastic cap on the scalp with 63 electrodes from BioSemi (Amsterdam, Netherlands) and added two electrodes behind the ears (mastoids). On the other hand, in another investigation, he applied the same apparatus with 32 electrodes placed on the scalp and 2 more electrodes behind the ears [31]. Additionally, 64 channels were employed by Wen [53] using SynAmps RT Amplifier from Neuroscan.

Additionally, Fronda et al. [42] used 15 channels located at Fp1, Fp2, F3, Fz, F4, T3, T4, C3, Cz, C4, P3, Pz, P4, O1, and O2 using LiveAMP from Brain Products (München, Germany). In this study, two biological signals were also captured: GSR using Biofeedback and HR. In addition, both Michael et al. [22] and Zoëga Ramsøy et al. [23] used B-Alert X10 EEG containing ten electrodes from ABM along with Tobii Pro X2-60 and Tobii ProGlasses2 eye trackers consecutively. Fewer electrodes were utilized by Savelli et al. [56]. They utilized three dry electrodes using MindWave Mobile2 from NeuroSky as well as a desk-mounted eye tracker using EyeTribe. Finally, in [40], the minimum number of electrodes was one, implanted on the forehead, utilizing the same previous device—MindWave Mobile2 from NeuroSky.

## 6. Discussion

The findings were analyzed based on two dimensions in order to answer our research questions: the systematic review (SR) and the challenges associated with neuro-tourism research.

### 6.1. SR Findings

Following the proposed classification scheme (Figure 4), we reviewed different perspectives in neuro-tourism research and found the following:

(1) When it comes to neuro-tourism applications, we found that destination was most commonly used in real-life applications. In contrast, the restaurant experience and pricing are the last considerations. Further, our findings indicated that the majority of neuro-tourism researchers employed eye-tracking rather than EEG.
(2) We also found that eliciting emotions (affect) through planned tasks were primarily applied to the tourist experience in terms of both stimuli and metrics.
(3) Regarding computational or statistical methods that can be used to analyze data, the majority of the studies (69.23%) employed statistical analyses such as ANOVA, *t*-test, mean, *p*-value, and others to compute statistical features such as mean and standard deviation, whereas ML models were applied in 30.77% of the studies.

There is a number of review articles covering the existing literature on neuro-tourism topics as noted in Section 5. These articles help advance knowledge in the neuro-tourism field and identify potential research areas to explore next. We anticipate a growing number of empirical research compared to review research.

### 6.2. Challenges and Future Directions

This systematic review validated the usefulness of neuro-tourism modalities for documenting the emotional responses of tourists and more solid and trustworthy results for assessing the decision-making of tourists when paired with self-report evaluations. However, this field is not free from difficulties. The following paragraphs discuss the challenges, limitations, and future directions of neuro-tourism.

The first major barrier in neuro-tourism is the technology associated with its modalities such as ET, EEG, GSR, or FAC. The development of these hardware sensors is improving in terms of size, portability, system usability, and sensory strength. However, researchers still require guidance and training when dealing with the device for the first time, particularly for EEG devices.

Research needs studies to examine different modalities evenly. We discovered most researchers employed ET, while other neuro-tourism techniques, including EGG and head and body movement, need additional research. The price of these technologies may be the cause, but the value of the outcomes obtained will outweigh its cost in the long run. We also anticipate greater study on all various neuro-tourism uses. For instance, tourists’ emotions in pricing and restaurant experience were investigated the least, yet these applications are not less important than others.

Additionally, selecting the most powerful stimuli in neuro-tourism studies is important. We discovered that the majority of researchers (87.18%) utilized various visual-based elicitation stimuli for all applications, while other researchers (12.82%) used predefined task stimuli mostly to investigate the tourist experience. However, there are no specific guidelines that determine the most effective stimuli except that the more novel the stimulus is, the more it influences memory [17] by engaging with more of a tourist’s senses.

The complex nature of human emotions makes it challenging to research several facets of tourists’ emotional responses at one time. Therefore, we recommend employing a triangulation (hybrid) method—a combination of more than one neuro-tourism modality with self-reports—to investigate errors especially if the research wants to evaluate multiple numbers of emotional tourists.

In addition, neuro-tourism is interdisciplinary. Experts from a variety of domains in neuroscience, engineering, computer science, psychology, and tourism are needed to create a successful neuro-tourism framework, select the appropriate modality, and effectively identify targeted emotions by choosing the suitable stimuli type and design. On the other hand, in the future, we anticipate more researchers collaborating in neuro-tourism research which presents the opportunity to advance the subject.

One major issue in neuro-tourism research is the lack of datasets. All previous studies that we analyzed had datasets from their experiments. We expect more effort in this regard in the future to facilitate building AI models based on emotional benchmark datasets specialized in neuro-tourism.

We also found that earlier research lacked an implementation of machine learning models, with most of them depending on statistical analyses (69.23%). Consequently, having public neuro-tourism databases will allow future research projects use ML models in systems for identifying emotions in tourists.

Lastly, because the neuro-tourism industry is still developing, the research is currently relatively fragmented. Each empirical research used its modalities differently than others, choosing different sample sizes, investigating diverse aspects of emotions (nonuniformed criteria for measuring emotions), and nonuniformed criteria for choosing stimuli, making it impossible to compare and generalize findings among studies.

Therefore, we anticipate further research that investigates rules and procedures for various use cases. We also anticipate strong conclusions in the next years when the same emotional responses of tourists are examined with various modalities on different populations, but with the same sample sizes using both identical and other standardized stimuli.

## 7. Conclusions

Recently, neuro-tourism has appeared as the application of neuroscience techniques to improve marketing tactics in the tourism industry [4]. With neuro-tourism, we can extract in-the-moment information from the subconscious minds (subjective responses) of visitors, such as emotions underpinning their decisions and attitudes toward tourism-related goods and services. Examples include traveler decisions on various destinations, tourist assessments of how appealing tourism-related products are, tourist opinions regarding the cost of service, tourist satisfaction with accommodations, etc. The goal is to provide tourism services and goods based on the actual tourist subconsciously generated thoughts. Such data cannot be gathered using conventional marketing strategies, e.g., surveys. The results are providing individualized tourism products and achieving better tourist satisfaction.

In this study, we systematically analyzed published articles on recognizing emotions in the tourism industry between January 2010 and September 2022. We have developed classifications, discussed the current literature, and provided an analysis for future work. We extracted 52 neuro-tourism articles from 32 different peer-reviewed journals. We read each article and then analyzed and classified them following our classification schema. The novelty of the neuro-tourism field has required both review papers and empirical studies to stand out. Yet, there is a serious need for future research.

Our findings found different neuro-tourism modalities used in the literature, such as EEG, ET, HR, GSR, head and body movements, etc. The current advancements in neuroimaging devices open the door for innovations in detecting tourists’ direct emotions and improving services provided in the tourism sector. Based on the findings of this review, we found limitations in using AI methods with neuro-tourism modalities. We anticipate future ML applications for detecting tourist emotions. Such applications will have benefits for both tourists and tourism agencies.

## Figures and Tables

**Table 1:** Applications in neuro-tourism.

Application	No. of Articles (%)	References
Destination	14 articles (35%)	[20,21,22,23,24,25,26,27,28,29,30,31,32,33]
Advertisement Design	7 articles (17.9%)	[29,34,35,36,37,38,39]
Accommodation	7 articles (17.9%)	[40,41,42,43,44,45,46]
Tourist Experience	6 articles (15.4%)	[47,48,49,50,51,52]
Tourism Risk	3 articles (7.7%)	[53,54,55]
Restaurant Experience	1 article (2.6%)	[56]
Pricing	1 article (2.6%)	[57]

**Table 2:** Neuro-tourism modalities; eye-tracking (ET), electroencephalography (EEG), galvanic skin response (GSR), facial-action-coding (FAC), heart rate (HR).

Modality	No. of Articles	References
ET	21	[24,26,27,28,29,30,32,35,36,37,38,39,41,43,44,45,46,52,54,55,58]
EEG	6	[21,31,40,47,48,53]
ET + EEG	3	[22,23,56]
GSR	2	[33,51]
GSR + Other Modalities	5	With EMG [20], With FAC [25], With HR [34], With EEG and HR [42], With EEG, ET, and HR [57].
FAC	1	[50]
Head and Body Motions + ET	1	[49]

**Table 3:** Emotion stimuli in neuro-tourism.

Emotion Stimuli	No. of Articles	% of Articles
Visual-based elicitation using images	12	30.77%
Audio–visual elicitation using short film video clips	5	12.82%
Multi-visual-based elicitation using images with text	5	12.82%
Planned task	5	12.82%
Visual-based elicitation using a website	4	10.26%
Multi-visual-based elicitation using videos, images, and text	2	5.13%
Visual-based elicitation using guidebooks	2	5.13%
Visual-based elicitation using text	2	5.13%
Multi-visual-based elicitation using videos and images	1	2.56%
Multiple techniques (true site visit + images)	1	2.56%

**Table 4:** Neuro-tourism metrics.

Measurement	Metric	References	Modality
Affect (emotion)	Arousal	[23,34]	ET/ GSR/ EMG/ EEG
	Valence	[40,42,49]	
	Arousal–valence	[20,21,22,25,31,33,47,51,52,57]	
	Satisfaction	[49,50]	FAC
	Trustworthiness	[45]	ET
	Anxiety	[47]	EEG
	Aesthetic perception	[26,28,48]	EEG/ET
	Like/dislike by ERP	[53]	EEG
Attention	The first view, time to the first fixation, fixation duration, fixation count, and gaze plots	[22,24,26,27,28,29,30,32,34,35,36,37,38,39,41,43,44,46,54,55,56,57,58]	ET/GSR
Cognitive load	Working memory	[22,23,56,57]	EEG
		[37]	ET

**Table 5:** Computation methods in neuro-tourism studies.

Method			No. of Articles	References
Preprocessing		Filtering	3	[51,53,56]
		Standardization	2	[20,40]
		Mean square	1	[42]
		Normalization	1	[22]
Feature Extraction		Principal component analysis (PCA)	2	[52,57]
ML Models (12 Studies, 30.77%)	Classification	Recurrent neural network with long short-term memory (RNN-LSTM) for 3-class classification: positive, negative, and neutral	1	[49]
	Regression	Linear regression analyses	5	[26,28,32,45,51]
		Structural equation modeling (SEM)	2	[50,57]
		Multiple regression analysis	1	[52]
		Neural network-based model	1	[23]
		Residual sum of squares	1	[48]
		Mixed logit	1	[30]
Statistical Models (27 Studies, 69.23%)		Analysis of variance (ANOVA)	15	[20,22,25,32,34,36,37,38,42,44,45,53,54,55,58]
		T-test	8	[21,31,35,40,41,45,46,51]
		Mean and standard deviation	20	[21,26,28,29,30,31,34,35,36,37,38,40,44,45,47,48,54,55,58]
		Mean	7	[20,24,25,39,43,46,56]
		*p*-value	9	[25,29,31,32,35,37,43,46,51]
		U test	4	[41,43,45,45]
		Repeated-measures ANOVA	4	[27,35,47,52]
		Chi-square test	3	[40,41,44]
		Correlation coefficients	2	[26,48]
		Bayes factor	1	[40]
		Cluster-based random permutation	1	[31]
		Spearman’s correlations	1	[47]

**Table 6:** Hardware devices used in neuro-tourism studies.

Modality	Devices	No. of Studies	References
EEG	BioSemi (Amsterdam, Netherlands)	2	[21,31]
	B-Alert X10 EEG brain scanner from ABM(Carlsbad, CA, USA)	2	[22,23]
	MindWave Mobile2 from NeuroSky (San Jose, CA, USA)	2	[40,56]
	ThinkGear ASIC Module from NeuroSky	1	[48]
	SynAmps RT 64-channel Amplifier from Neuroscan	1	[53]
	EPOC from Emotiv Insight (San Francisco, CA, USA)	1	[47]
	LiveAMP 15-channel EEG from Brain Products (München, Germany)	1	[42]
ET	Tobii Pro X2-60 Eye Tracker (Tobii Technology, Inc. Stockholm, 379 Sweden)	5	[22,32,36,38,44]
	Tobii ProGlasses2 wearable eye tracker	3	[23,24,39]
	Eyelink1000 desktop eye tracker	3	[35,37,52]
	EyeTribe desk-mounted eye tracker	2	[45,56]
	SMI Red 500 remote system (SensoMotoric Instruments, Berlin, Germany)	2	[28,41]
	Eyeso Ec80 eye movement instrument	2	[54,55]
	Gazepoint GP3 HD	1	[29]
	Tobii	2	[26,43]
	Tobii T120	2	[30,58]
	Tobii X1L	1	[27]
	Tobii X2-30	1	[46]
	Pupil Labs eye tracker	1	[49]
GSR	BiopacTM BioNomadix hardware (wearable wireless devices)	2	[20,25]
	Empatica E4 wristband	1	[51]
	Biofeedback device (Biofeedback 2000 x-pert, version 7.01, Schuhfried GmbH, Mödling, Austria)	1	[42]
	Two standard AG/AGCL electrodes	1	[34]
Head and Body Motions	SenStick sensor board	1	[49]

## Data Availability

Not applicable.

## Appendix A. Neuro-Tourism Article Classification by Source Titles

The 52 neuro-tourism articles were analyzed from 32 different peer-reviewed journals. Most of the journals were related to tourism and hospitality research. Table A1 documents journals with more than one published article.

**Table A1:** Article classification by most-used journals.

Source Title Name	No. of Articles
*Current Issues in Tourism*	7
*Journal of Hospitality and Tourism Research*	4
*International Journal of Contemporary Hospitality Management*	3
*Journal of Travel Research*	3
*Annals of Tourism Research*	2
*Journal of Destination Marketing and Management*	2
*Journal of Vacation Marketing*	2
*Neuroquantology*	2
*Tourism and Hospitality Research*	2
*Tourism Management*	2
*Tourism Recreation Research*	2
